# Non-invasive Imaging of Endothelial Progenitor Cells in Tumor Neovascularization Using a Novel Dual-modality Paramagnetic/Near-Infrared Fluorescence Probe

**DOI:** 10.1371/journal.pone.0050575

**Published:** 2012-11-30

**Authors:** Xin-Yi Wang, Shenghong Ju, Cong Li, Xin-Gui Peng, Alex F. Chen, Hui Mao, Gao-Jun Teng

**Affiliations:** 1 Jiangsu Key Laboratory of Molecular and Functional Imaging, Department of Radiology, Zhongda Hospital, Medical School, Southeast University, Nanjing, Jiangsu, China; 2 Key Laboratory of Smart Drug Delivery, Ministry of Education & PLA, School of Pharmacy, Fudan University, Shanghai, China; 3 Department of Surgery, University of Pittsburgh School of Medicine, Pittsburgh, Pennsylvania, United States of America; 4 Department of Radiology and Imaging Sciences, Emory University School of Medicine, Atlanta, Georgia, United States of America; Northwestern University, United States of America

## Abstract

**Objective:**

Bone-marrow derived endothelial progenitor cells (EPCs) play an important role in tumor neovasculature. Due to their tumor homing property, EPCs are regarded as promising targeted vectors for delivering therapeutic agents in cancer treatment. Consequently, non-invasive confirmation of targeted delivery via imaging is urgently needed. This study shows the development and application of a novel dual-modality probe for *in vivo* non-invasively tracking of the migration, homing and differentiation of EPCs.

**Methods:**

The paramagnetic/near-infrared fluorescence probe Conjugate **1** labeled EPCs were systemically transplanted into mice bearing human breast MDA-MB-231 tumor xenografts. Magnetic resonance imaging (MRI) and near-infrared (NIR) fluorescence optical imaging were performed at different stages of tumor development. The homing of EPCs and the tumor neovascularization were further evaluated by immunofluorescence.

**Results:**

Conjugate **1** labeled EPCs can be monitored *in vivo* by MRI and NIR fluorescence optical imaging without altering tumor growth for up to three weeks after the systemic transplantation. Histopathological examination confirmed that EPCs were recruited into the tumor bed and then incorporated into new vessels two weeks after the transplantation. Tumor size and microvessel density was not influenced by EPCs transplantation in the first three weeks.

**Conclusions:**

This preclinical study shows the feasibility of using a MRI and NIR fluorescence optical imaging detectable probe to non-invasively monitor transplanted EPCs and also provides strong evidence that EPCs are involved in the development of endothelial cells during the tumor neovascularization.

## Introduction

The fact that tumor growth and metastasis depend on neovascularization promotes extensive investigations on its role in tumor progression [1]. Recent studies indicate that neovascularization includes two mechanisms: angiogenesis and vasculogenesis. The former refers to the proliferation of endothelial cells (ECs) to give rise to preexisting vessels, while the latter describes the process by which the primitive progenitors are recruited and differentiate *in situ* to develop new vessels [2]. Earlier studies have demonstrated the contribution of the bone marrow derived endothelial progenitor cells (EPCs) in tumor neovascularization [3]–[5]. The paracrine signals from a growing tumor initiates the activation, mobilization and recruitment of EPCs to the tumor site [2], [6]. It is suggested that EPCs contribute to neovascularization either through incorporating into vessels or by secreting pro-angiogenic cytokines [7]. Thus, the recruitment of EPCs by a tumor may accelerate neovascularization, provide tumor tissues with more nutrients and ultimately promote tumor growth. Bagley et al. demonstrated that co-injection of EPCs and tumor cells systemically resulted in an increased rate of tumor growth [8]. Furthermore, tumor growth can be inhibited by blocking the recruitment of the bone marrow derived EPCs [5], [9]. Thus, transplantation of EPCs can potentially accelerate tumor growth. However, results from others only showed a minor or no contribution of EPCs to neovasculature [10], [11]. The main reason for this discrepancy might be the different tumor types and tumor growth stages used in the different studies. The differences in phenotype characterization of EPCs and the confusion of EPCs with other perivascular cells have also been suggested as possible reasons of different observations by previous reports [4], [6]. Such inconsistent results, along with highly complex mechanism involved in the tumor vascularization, promote extended investigations to further understand the function of EPCs in the neovascularization and tumor development.

Given their tumor homing properties, EPC carrying imaging probes have been applied to visualize and monitor tumor development in recent studies [12], [13]. Furthermore, EPCs are investigated as targeted cellular vectors to carry suicide genes or chemotherapeutics to treat cancers [14], [15]. Therefore, EPCs provide a promising platform for delivering the therapeutics and/or imaging probes for imaging directed cancer treatment, which is desirable in the clinical setting. Among all imaging modalities for tumor imaging and cell tracking, magnetic resonance imaging (MRI) is known for its advantages in high spatial resolution, good soft tissue contrast, three-dimensional imaging depth and not depending on radioactive tracers. Furthermore, its preclinical discoveries and development can be easily translated into the clinical applications. However, the major limitation of MRI in molecular imaging applications is its suboptimal detecting sensitivity. On the other hand, optical imaging is sensitive, cost-effective and convenient for the chemical manipulation. In particular, the near-infrared (NIR) fluorescence (650 to 900 nm), compared to the visible light, is capable of penetrating deeper tissue and reducing background auto fluorescence because the absorbance of endogenous molecules, such as water and hemoglobin, are low in NIR range [16]–[18]. Furthermore, the NIR fluorescent imaging has been clinically available in recent years [19]. Therefore, the complementary strengths from magnetic resonance (MR) and optical imaging can provide a dual-modality imaging solution for preclinical investigations [17], [20].

In the previous work, we have shown a novel dual-modality imaging probe, bacterial cytosine deaminase poly-_L_-lysine (bCD-PLL)-Gd^3+^-Cy5.5-rhodamine named Conjugate **1**, with high longitudinal relaxivity R_1_ for MRI contrast enhancement and strong fluorescence signal for NIR imaging, as well as low cytotoxicity and efficient cell uptake by EPCs without compromising the cell viability [21]. Gd^3+^ incorporated in Conjugate **1** provides MRI contrast for in vivo cell tracking and monitoring using T_1_-wighted MRI, while fluorophore, rhodamine enables tracking the probe molecules in cells under fluorescence microscope. In addition, Cy5.5 can be used for *in vivo* NIR optical imaging and histological studies to validate MRI results. Bacterial cytosine deaminase (bCD), a therapeutic enzyme in cancer gene therapy, has been shown to increase cellular uptake rate after being conjugated with poly-_L_-lysine (PLL) [22], [23].

In this study, we report a new strategy for non-invasively visualization of the homing, migration and differentiation of EPCs in vivo. We demonstrated the transcardiac transplanted EPCs can be tracked in a xenograft tumor model by MR and optical imaging for up to three weeks without altering the tumor growth. Subsequent histopathological examination confirmed that delivered EPCs were recruited to the tumor bed and then incorporated into new vessels.

## Materials and Methods

### Synthesis of Conjugate 1

bCD-PLL was prepared according to the synthetic procedure as previously described [24]. Briefly, bCD protein was isolated from transformed Escherichia coli transfected with the bCD vector. Treatment of NHS esters of DOTA, Cy5.5 and rhodamine with PLL, respectively, yielded a bCD-PLL-DOTA-Cy5.5-rhodamine complex. Gd^3+^ was then incorporated into the complex by chelating with DOTA. The conjugation of bCD and PLL in the complex with both MRI (Gd^3+^) and optic imaging (Cy5.5 and rhodamine) reporters then provided a functionalized probe. A narrow molecular size distribution was detected by dynamic light scattering with an average hydrodynamic radius of 9.4 nm and the molecular weight of Conjugate **1** was measured as 345 kDa [24].

### Isolation and Culture of EPCs from Bone Marrow

All animal experiments in this study were approved by the Institutional Animal Use and Care Committee of Southeast University. All surgical procedures were done under sodium pentobarbital anesthesia. All efforts were done to minimize suffering. As a relative large number of EPCs were needed in this study, rats were selected as the cell donator instead of mouse for its abundant amount of bone marrow. Bone marrow was harvested from the tibias and femurs of 10 male Sprague–Dawley rats (4-week old, Academy of Military Medical Science, Beijing, China) and resuspended with PBS. The suspension was added to lymphocyte separation medium (HaoYang Biological Manufacture Co., Tianjin, China) and centrifuged at 400 g at room temperature for 30 minutes. Mononuclear cells (MNCs) buffy coat was isolated and washed twice with PBS by centrifuging at 200 g for 10 minutes. MNCs were cultured with endothelial basal medium (Lonza, Basel, Switzerland) supplemented with endothelial cell growth medium SingleQuots containing 5% fetal bovine serum, vascular endothelial growth factor (VEGF), human fibroblast growth factor B, human epidermal growth factor, insulin-like growth factor 1, ascorbic acid, hydrocortisone, and GA-1000 (Lonza). Cells were seeded on 25 cm^2^ fibronectin (Sigma-Aldrich, St. Louis, MO)-coated culture flasks (Corning Inc., NY) at 37°C, 5% CO_2._ The culture medium was changed every 3–4 days. Cells were maintained for four passages and then used for transplantation. To characterize the phenotype, cells were incubated with DiI-labeled acetylated low-density lipoprotein (DiI-Ac-LDL; Invitrogen Corporation, Carlsbad, CA) for 4 h at 37°C. Lectin binding was analyzed using fluorescein isothiocyanate (FITC)-labeled Ulex europaeus agglutinin I (FITC-UEA I; Invitrogen Corporation) [25]. The expression of various progenitor and endothelial lineage markers such as CD34, CD133, CD31 (Santa Cruz Biotechnology Inc., Santa Cruz, CA) and VEGFR-2 (abcam Biochemicals, Cambridge, UK) were analyzed by immunocytochemistry. PBS without primary antibodies was served as negative controls and cell nuclei were stained with 4′,6-diamidino-2-phenylindole (DAPI; Beyotime Institute of Biotechnology, Haimen, China). To confirm the purity of the cultured cells, flowcytometry was done to assess the levels of CD34, CD133, CD31 (Santa Cruz Biotechnology Inc) and VEGFR-2 (abcam Biochemicals) using a FACSscan instrument (FACSCalibur, BD Biosciences, NJ).

### Breast Cancer Xenograft Models

VEGF-overexpressing human breast MDA-MB-231 cancer cells were purchased from American Type Culture Collection (ATCC, Manassas, VA) and were passaged for fewer than six months. Cells were maintained in RPMI-1640 medium supplemented with 10% fetal bovine serum (KeyGen Biotech Co., Nanjing, China) and harvested when they reached 80% confluence to maintain exponential growth. MDA-MB-231 cells were inoculated at a concentration of 1×10^6^ in 0.1 mL RPMI-1640 medium in the upper right thoracic mammary fat pad of female BALB/c nude mice (5-week old, Academy of Military Medical Science). Tumors were palpable at 7 days after implantation and reached a volume of approximately 20 mm^3^ when they were used for study.

### Labeling of Endothelial Progenitor Cells and Cell Transplantation

EPCs were incubated with 2 µM Conjugate **1** for 24 hours [21]. Adherent cells were washed 3 times with PBS and then detached using 0.25% trypsin-EDTA (KeyGen Biotech Co.). Once the tumor xenograft model was established, Conjugate **1** labeled EPCs (2×10^6^ cells) were transplanted in animals in the group 1 (*n* = 21) via intracardiac transplantation. Animals in the group 2 (*n* = 21) were transplanted with unlabeled EPCs. Animals in the group 3 (*n* = 21) were transplanted with 0.1 mL culture medium. EPC labeling efficiency with Conjugate **1** was calculated using an inverted phase-contrast microscopy (Axioscop; Zeiss Co. Ltd., Oberkochen, Germany) by counting rhodamine positive cells from five different fields and expressed as the average percentage of these cells.

### Magnetic Resonance Imaging and Data Acquisition

*In vivo* MRI experiments were performed on a small bore 7 T MR scanner (Bruker PharmaScan; Bruker BioSpin, Karlsruhe, Germany) before and 1, 3, 5, 7, 10, 14 and 21 days after EPC transplantation. Three mice in each group were scanned at each time point. The details on experimental time points and group designation were shown in Table S1 and S2. During the MRI scan, animals were anesthetized with 1.0% isoflurane (Keyuan Pharmaceutical Co., Shandong, China) in a head holder through a nosecone and were monitored for respiration rate. T_1_-weighted spin-echo (repetition time/echo time, 500/15 msec) and T_2_-weighted fast spin-echo (repetition time/echo time, 3000/36 msec) sequences were used with 3×3 cm^2^ field of view (FOV) and 1 mm slice thickness, 20 slices prescribed from the apex to base of the tumor. Tumor volume of each animal was measured from T_2_ weighted images using Image J 1.41 software (NIH) at each time point. Tumor volume doubling time (VDT) was calculated as VDT = 14×log2/log (V_21_/V_7_), in which V_7 = _tumor volume at day7, V_21 = _tumor volume at day 21 [26]. A round region of interest (ROI) of approximately 10 mm^2^ was used for tumor signal intensity measurement. Contrast-to-noise ratio (CNR) was calculated as follows: CNR = (SI_T_-SI_M_)/SI_N_, in which SI_T_ *= *SI of tumor, SI_M_ = SI of muscle, and SI_N_ = SI of background noise.

To obtain maps of the longitudinal relaxation time T_1_, a set of images were collected using a rapid acquisition with relaxation enhancement sequence and the following parameters: TR = 200, 400, 800, 1500, 3000, 5000 ms, TE = 11 ms, flip angle = 180°, slice thickness 1 mm, matrix = 256×256, FOV = 3.0×3.0 cm. T_1_ relaxation time at each voxel was calculated using a nonlinear fitting algorithm with a exponential recovery function. Averaged intratumoral and hepatic T_1_ relaxation times of group 1 and group 2 at each time point were obtained from quantitative T_1_ maps.

### In Vivo and Ex Vivo Optical Imaging

At 1, 3, 5, 7, 10 and 14 days after EPC transplantation, MDA-MB-231 tumor-bearing nude mice in group 1 and group 2 (*n* = 3 per time point in each group) were subjected to optical imaging using Maestro *In-Vivo* Optical Imaging System (excitation = 675 nm, emission = 695 nm, exposition time 500 ms; Caliper Life Sciences, MA). The images from decubitus and lateral position of the same animal were acquired and analyzed using Maestro 2.4 software (Caliper Life Sciences). After animals were sacrificed, *ex vivo* images of the excised tumors, livers, spleens, kidneys and lungs were collected (exposition time 2000 ms) followed by histological analysis.

Fluorescence images consisting of auto fluorescence spectra and Cy5.5 spectra were then unmixed using multi-excitation spectral analysis functions. To measure the *ex vivo* fluorescence intensity of each organ, the ROIs were placed on the organ and the total signals were normalized by the exposure time and the area of ROI (scaled counts/s) [27].

### Histological Analysis and Immunofluorescence

After *in vivo* imaging studies, mice were sacrificed and immediately perfused with normal saline intracardially to prefix tissues. One half of the tumor tissues in group 1 and 3 were harvested and incubated in 4% paraformaldehyde for overnight, then preserved in a 20% sucrose solution at 4°C. The other half were prepared for quantitative analysis of gadolinium. Tissues embedded in OCT compound (Sakura Finetek USA, Inc., Torrance, CA) were snap frozen in the freezing microtome (Leica Microsystems GmbH, Wetzlar, Germany). Consecutive sections of 5-µm thickness were sliced and mounted on poly-L-lysine-coated glass slides.

To confirm the distribution of labeled EPCs in tumor sections with T_1_-weighted images, tumor tissue slides collected from the group 1 at day 1, 3, 5, 7 were scanned using a laser scanning confocal fluorescence microscope (Olympus FluoView FV1000; Olympus Optical Co. Ltd., Tokyo, Japan) immediately after slicing from the tumors. Slides from the group 1 and group 3 were then evaluated by immunofluorescent staining of rabbit anti-rat endothelial progenitor cell primary antibody CD34, CD133 (Santa Cruz Biotechnology Inc.), VEGFR-2 (abcam Biochemicals) and endothelial cell primary antibody CD31 (Santa Cruz Biotechnology Inc.) followed by staining with Alex Fluo 488-conjugated secondary goat anti-rabbit IgG (Invitrogen Corporation). PBS without primary antibodies was served as negative controls. The slides were observed using a laser scanning confocal fluorescence microscope (Olympus Optical Co. Ltd.) to detect the expression of endothelial progenitor cell or endothelial cell markers. To detect Cy5.5-positive cells in group 1 tumor, the excitation wavelength was set as 675 nm.

The numbers of Cy5.5 positive cells in tumor sections were calculated from five different fields of view (magnification×200) within each tumor at selected time points (Table S1). Tumor microvessel densities (MVD) were calculated by scanning serial sections stained with anti-CD31 antibody. CD31 positive vessels were counted in the double blind study from five different fields within each tumor and expressed as the average number of microvessels per square millimeter (mean ± SD) [28].

### Quantitative Analysis of Gd in Tissues

One half of tumor, liver, spleen, kidney and lung tissues unfixed with paraformaldehyde from group 1 were weighed and digested with nitric acid and hydrogen peroxide. Quantitative analysis of gadolinium in the tissue was performed using an inductively coupled plasma mass spectrometry (ICP-MS, PerkinElmer, Waltham, MA).

### Statistical Analysis

Statistical analyses were performed with the SPSS software (version 20; SPSS Inc., Chicago, IL). All numeric data are presented as mean ± SEM. For statistical comparisons, the independent-sample *t*–test and one-way ANOVA correlation and regression analysis were applied. ANOVA was performed followed by a least significant difference test. A value of *p*<0.05 was considered significant.

## Results

### EPC Culture and Cell Labeling

**Figure 1:**
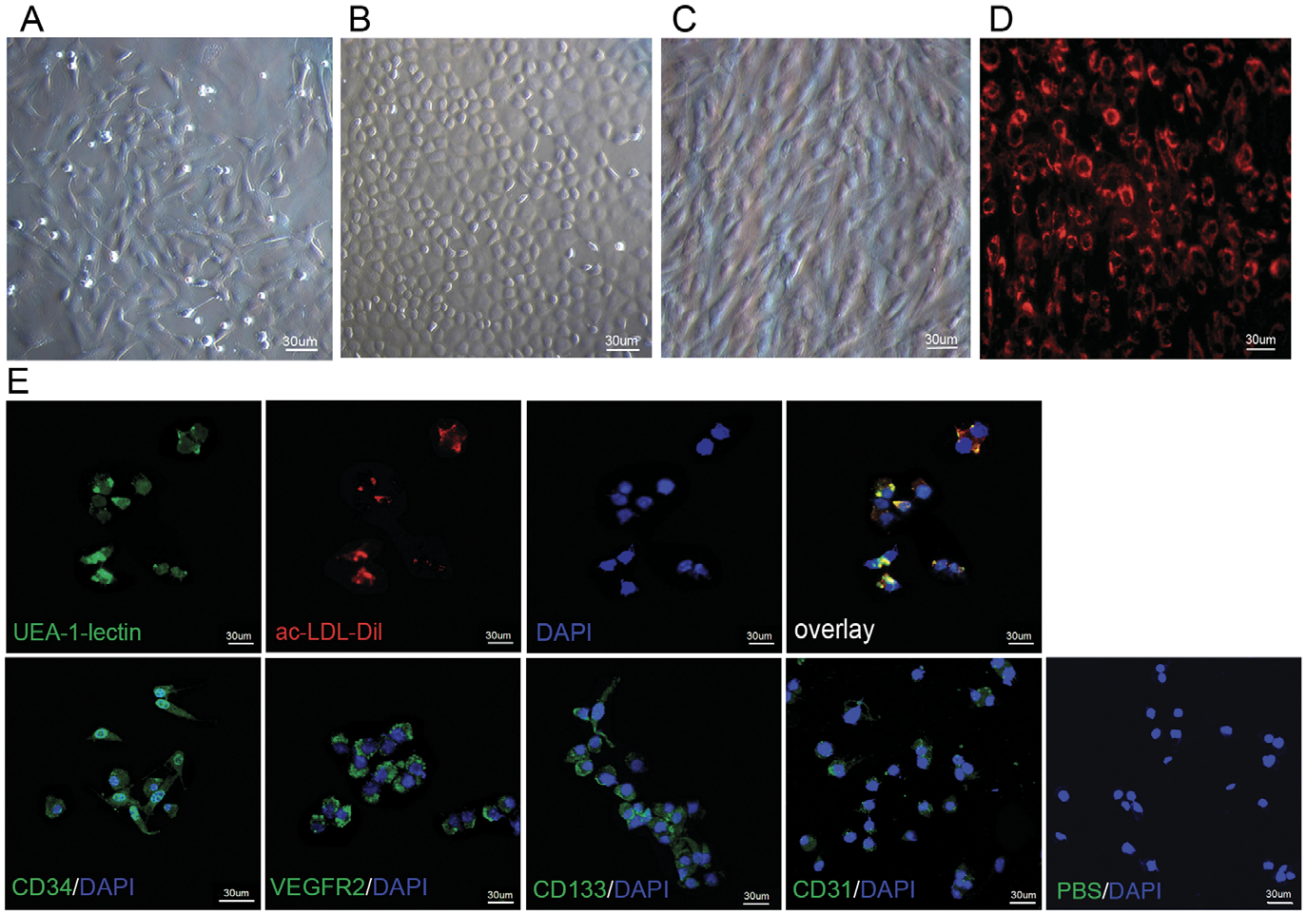
Cellular morphology of cultured EPCs. (A) The adherent cells showed irregular shape and began to form clusters 5 days after incubation. (B) A typical “cobblestone” appearance which is the characteristics of an endothelial cell monolayer was found at day 10. (C) Cells passaged 3 times at a ratio of 1∶2 with an average of 7 days of growth showed homogenous spindle shaped morphology. (D) Fluorescence microscopy revealed the uptake of Conjugate **1** by adherent cells with positive rhodamine signals. (E) These cells were stained positively with FITC-UEA I, DiI-Ac-LDL and other endothelial progenitor cell markers, and showed low expression of endothelial cell marker CD31.

Cellular morphology of cultured EPCs was observed with an inverted microscope. Freshly isolated MNCs appeared small and round. After 48 hours under the culture conditions used, cells showed signs of adherence. The adherent cells assumed irregular shapes and began to form clusters 5 days after incubation (Fig. 1A). A typical “cobblestone” appearance, which is the characteristics of an endothelial cell monolayer, was found at day 10 (Fig. 1B). Cells passaged 3 times at a ratio of 1∶2 with an average of 7 days of growth showed homogeneous spindle shaped morphology (Fig. 1C).

Cellular uptake and localization of the dual-modality imaging probe, Conjugate **1**, in EPCs were investigated by fluorescence microscopy. Fig. 1D shows fluorescence signals from the probes in the adherent cells, suggesting the positive uptake of Conjugate **1** by EPCs. Rhodamine fluorescence (*red*) was localized in small intracellular vesicles in the perinuclear region. Labeling efficiency with Conjugate **1** was (96.72±2.55) % for EPCs.

The cells that maintained for four passages were confirmed to be differentiating endothelial progenitor cells as they were stained positive with both FITC-UEA I and DiI-Ac-LDL. Additionally, these cells expressed EPC specific markers of CD34, VEGFR-2 and CD133. Low expression of endothelial cell marker CD31 was observed (Fig. 1E). FACS analysis revealed that 26.58% of adherent cells express CD34, 28.37% VEGFR-2, 32.39% CD133 and 0.78% CD31.

### MR Imaging and Contrast Enhancement from Labeled EPCs

**Figure 2:**
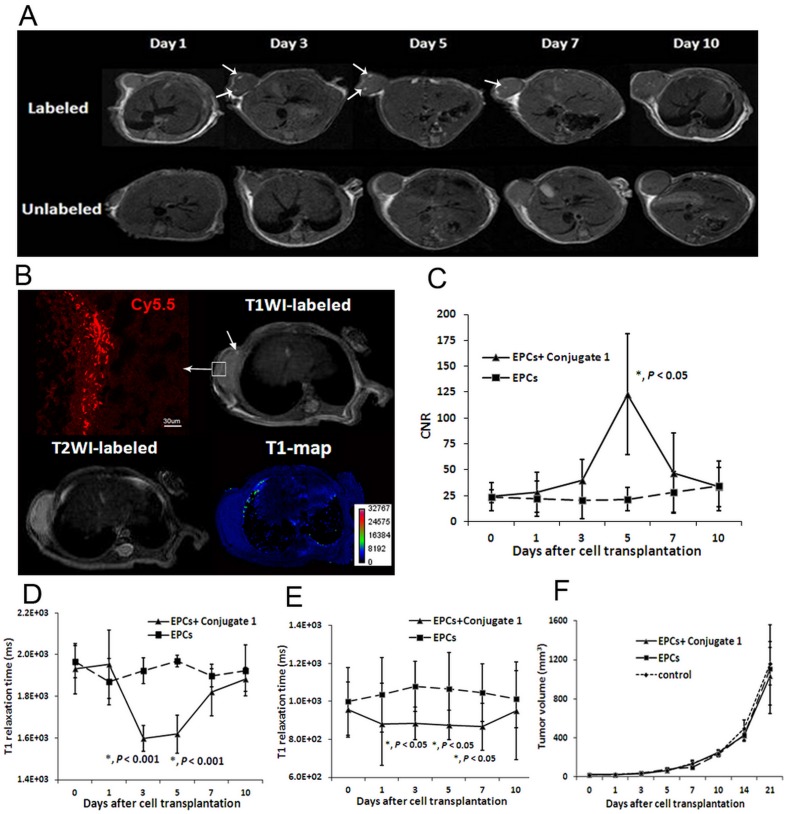
MRI exams demonstrated the homing of labeled EPCs into tumors. (A) Sprinkles of hyperintense region at the margin of the tumor (*arrows*) on T_1_-weighted images indicate the presence of Conjugate **1** labeled EPCs in the tumor as early as 3 days after transplantation. Significant hyperintense areas within the tumor were also observed at day 5 and day 7. (B) A representative T_1_-weighted image, T_2_-weighted image and pseudo-color T_1_ map obtained at day 5. Images of fluorescent microscopy showed that Cy5.5 positive cells were found at the corresponding areas of high signal intensity (*arrows*) on T_1_-weighted images. Scale bar = 30 µm (C) The CNR from the region of interest of group 1 reached the maximum at day 5. The difference between group 1 and 2 in CNR was statistically significant (*p*<0.05, *n* = 3). (D) Coinciding with the signal enhancement detected from T_1_-weighted images, intratumoral T_1_ relaxation time initially decreased to (1.60±0.06)×10^3^ ms at day 3 and persisted at (1.62±0.09)×10^3^ ms until day 5. The mean value recovered to the base line two days later. The significant decrease of T_1_ relaxation time (*p*<0.001, *n* = 3) confirmed the homing property of transplanted EPCs. (E) Although the accumulation of Conjugate **1** labeled EPCs in the liver did not induce significant contrast enhancement, a significant decrease of hepatic T_1_ relaxation time was observed from day 3 to day 7 (*p*<0.05, *n* = 3). (F) During the three weeks of monitoring period, no significant difference in tumor volume was found between each group at each time point (*p*>0.05, *n* = 3).

Sprinkles of hyperintense signals were observed on T_1_-weighted images (*arrows*) in the tumors in group 1 with Conjugate **1** labeled EPCs. The signal intensities of tumors in group 2 were moderate and almost homogeneous (Fig. 2A). This result clearly demonstrated the presence of labeled EPCs in tumors after systemic delivery. The signal changes appeared in the margin of the tumor as early as 3 days after cell transplantation. Heterogeneous hyperintense areas were also observed within the tumor at day 5 after cell transplantation, suggesting the intratumoral delivery of transcardiac administered labeled EPCs. The hyperintense signal became weaker at day 7 and almost disappeared at day 10 of the post EPC transplantation. Fig. 2B shows representative T_1_-weighted and T_2_-weighted images as well as pseudo-color T_1_ map of a mouse obtained at day 5. Laser scanning confocal microscopy showed Cy5.5 positive cells at the site of the tumor where the high signal intensity on T_1_-weighted images was observed correspondingly. The CNR from the ROIs of the tumors in group 1 also reached a maximum at day 5. The CNR difference between group 1 and 2 was statistically significant at day 5 (122.87±58.59 vs. 21.71±11.02, *p*<0.05, *n = *3, Fig. 2C). No significant difference was detected after day 7.

The intratumoral T_1_ relaxation time were obtained before and after the transplantation of the Conjugate **1** labeled EPCs. Coincident with T_1_-weighted images, a significant decrease of intratumoral T_1_ relaxation time was observed at day 3, comparing to that of group 2 [(1.60±0.06)×10^3^ ms vs. (1.92±0.06)×10^3^ ms, *p*<0.001, *n = *3, Fig. 2D]. The trend of T_1_ decreasing persisted until day 5 [(1.62±0.09)×10^3^ ms vs. (1.97±0.03)×10^3 ^ms, *p*<0.001, *n* = 3, Fig. 2D]. The mean value of T_1_ recovered to (1.82±0.11)×10^3^ ms at day 7, and then was gradually reach to the baseline. A maximum reduction of 17.78% in the averaged T_1_ relaxation time was found at 5 days after transplantation of labeled cells. The significant decrease of the T_1_ relaxation time in the quantitative T_1_ map contributed to the signal enhancement detected from T_1_-weighted images as well as the highest CNR at day 5, confirming the homing of transplanted EPCs. Furthermore, a significant decrease of hepatic T_1_ relaxation time was observed from day 3 to day 7 (*p*<0.05, *n = *3, Fig. 2E), indicating its phagocytosis of transplanted cells. The necrosis and hemorrhage in the center of the tumor were initially detected from T_2_-weighted images at day 10. Therefore, the CNR and intratumoral T_1_ relaxation time were not calculated after day 10 in consideration of the interference of the tumor growth related signal changes.

The averaged tumor volumes of each group were calculated at each time point. The tumor volume increased from 20.62±3.85 mm^3^ to 1031.89±295.83 mm^3^ at 21 days after transplantation of Conjugate **1** labeled EPCs in group 1, while the averaged tumor volumes of group 2 and 3 increased from 19.47±6.51 mm^3^ and 18.65±4.29 mm^3^ to 1105.34±453.82 mm^3^ and 1161.58±223.66 mm^3^, respectively. Tumor volumes in all three groups increased more than 50 folds after three weeks. However, no significant difference in the tumor volume was found between each group at each time point (*p*>0.05, *n* = 3, Fig. 2F). No statistical significant difference between VDTs of group 1, 2, 3 (4.25±0.94 days vs. 4.68±0.87 days vs. 4.74±0.19 days, *p*>0.05, *n* = 3) was observed.

### In Vivo and Ex Vivo Optical Imaging Studies

**Figure 3:**
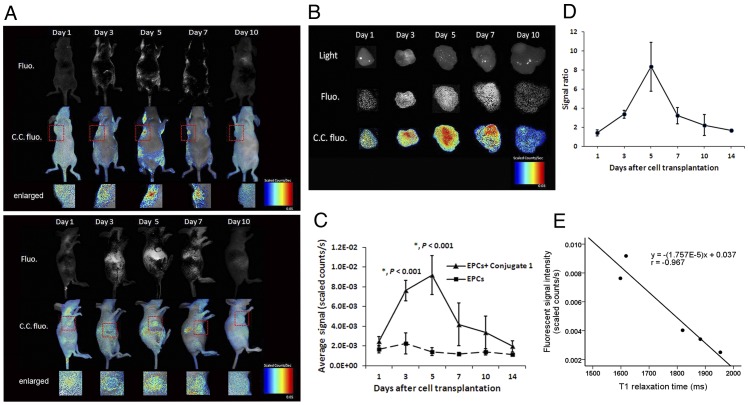
Enhancement of fluorescence intensity at the tumor area in optical imaging. (A) Near infrared fluorescence images and color-coded fluorescence images of decubitus and lateral position of a mouse post transplantation show peak signal intensity appeared at day 5 (Fluo, fluorescence; C.C. fluo, color-coded fluorescence). (B) A strong Cy5.5 fluorescence emission was detected in the tumor tissue 5 days after EPC transplantation and remained until day 7. (C) The peak value of average fluorescent signal intensity was measured at day 5 with (9. 18±1.98)×10^−3^ scaled counts/s. Signal intensity changes from day 3 to day 5 in group 1 were statistically significant (*p*<0.001, *n* = 3). (D) The signal ratio of tumors (signal ratio was calculated as signal intensities of the tumor with labeled EPCs over the control tumor with unlabeled EPCs) reached the maximum of 8.34±2.55 at day 5. (E) A linear correlation between averaged fluorescent signal intensity and intratumoral T_1_ relaxation time was observed (*r* = −0.967, *p*<0.01).

The homing of Conjugate **1** labeled EPCs to the tumor resulted in fluorescence intensity enhancement in the tumor area observed in optical imaging. Fig. 3A shows a representative NIR fluorescence images and color-coded fluorescence images of a mouse from group 1 at the decubitus and lateral positions after transplantation of labeled EPCs. Intratumoral homing of Conjugate **1** labeled EPCs was demonstrated with the presence of NIR fluorescence signals around the tumor (red dashed lined) as early as 3 days after cell transplantation. The signal intensity appeared to reach the peak at day 5 and then persisted until day 7 before disappeared after day 10. Due to the interference of fluorescence signal from the labeled EPCs trapped in the liver, absolute quantification of labeled cells with fluorescence signal was not performed.

To further verify the EPC homing as observed *in vivo*, *ex vivo* imaging was performed on the excised tumors (Fig. 3B). A strong Cy5.5 fluorescence was detected in the tumor tissue 5 days after EPC transplantation, which was consistent with MRI data. However, the fluorescent signal remained until day 7, while MRI contrast almost disappeared at this time point.

Time-dependent changes of the intratumoral fluorescence intensity was also investigated using the *ex vivo* images of excised tumors. The peak value of average fluorescent signal intensity of the tumor was found at day 5 with (9.18±1.98)×10^−3^ scaled counts/s. Signal intensity changes from day 3 and day 5 in group 1 were statistically significant (*p*<0.001, *n* = 3, Fig. 3C) while the signal ratio (signal ratio was calculated as signal intensities of the tumor with labeled EPCs over the control tumor with unlabeled EPCs) reached a maximum of 8.36±2.55 at day 5 (Fig. 3D). A linear correlation between the averaged fluorescent signal intensity from the optical imaging and intratumoral T_1_ relaxation time from MRI was observed (*r* = −0.967, Fig. 3E) at time points of 1 to 10 days after transplantation of labeled EPCs.

**Figure 4:**
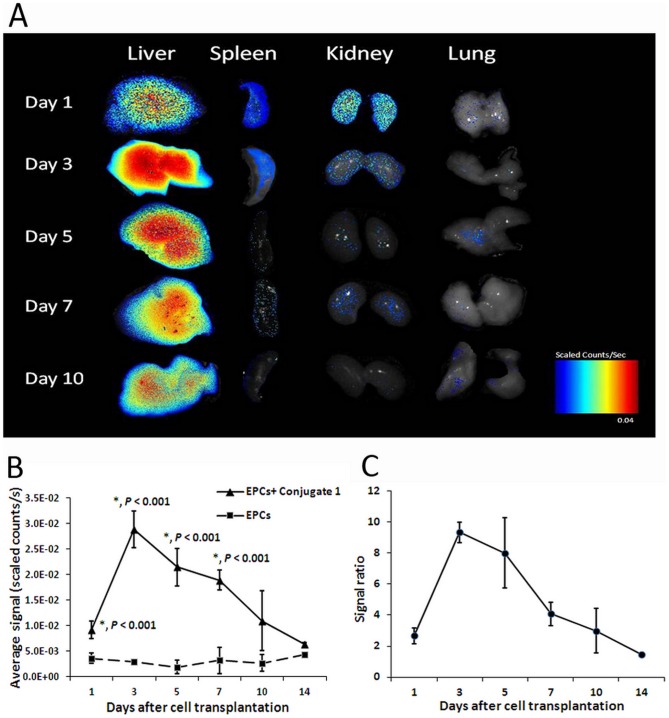
*Ex vivo* NIR imaging of other organs. (A) Color-coded fluorescence images indicated that strong signal enhancement in the liver persisted from day 3 to day 7. Weak signals were detected from other organs at day 1 and 3. (B) The levels of liver signal intensity of group 1 and 2 were shown. Significant fluorescence intensity differences in the liver between group 1 and 2 were observed from day 1 to day 7 (*p*<0.001, *n* = 3). The peak value was detected at day 3. (C) The liver signal ratio between group 1 and 2 was 9.34±0.68 at 3 days post transplantation of EPCs.

Due to the reticuloendothelial system (RES) that has phagocytic function, amounts of transplanted EPCs in RES is naturally higher, in comparison to that in the tumor. To investigate the distribution of transplanted EPCs in other organs, livers, spleens, kidneys and lungs were also examined *ex vivo* using NIR imaging. It was found that the liver appeared to have the highest uptake of transplanted cells. The strong signal enhancement persisted in the liver from day 3 to day 7 (Fig. 4A). Weak signals were also detected from spleen and kidney at day 1 and 3. Significant differences of liver fluorescent signal intensities between group 1 and 2 were observed from day 1 to day 7 (*p*<0.001, *n* = 3). The maximum liver signal [(28.84±3.55)×10^−3^ scaled counts/s] in group 1 was detected at day 3 after EPC transplantation (Fig. 4B) while the signal ratio was as high as 9.34±0.68 (Fig. 4C). Afterwards, signal intensity in the liver decreased gradually. The variation tendency of liver fluorescence intensity in group 1 was similar to the T_1_ relaxation time measured in MRI.

### Histology and Immunofluorescence Validation

**Figure 5:**
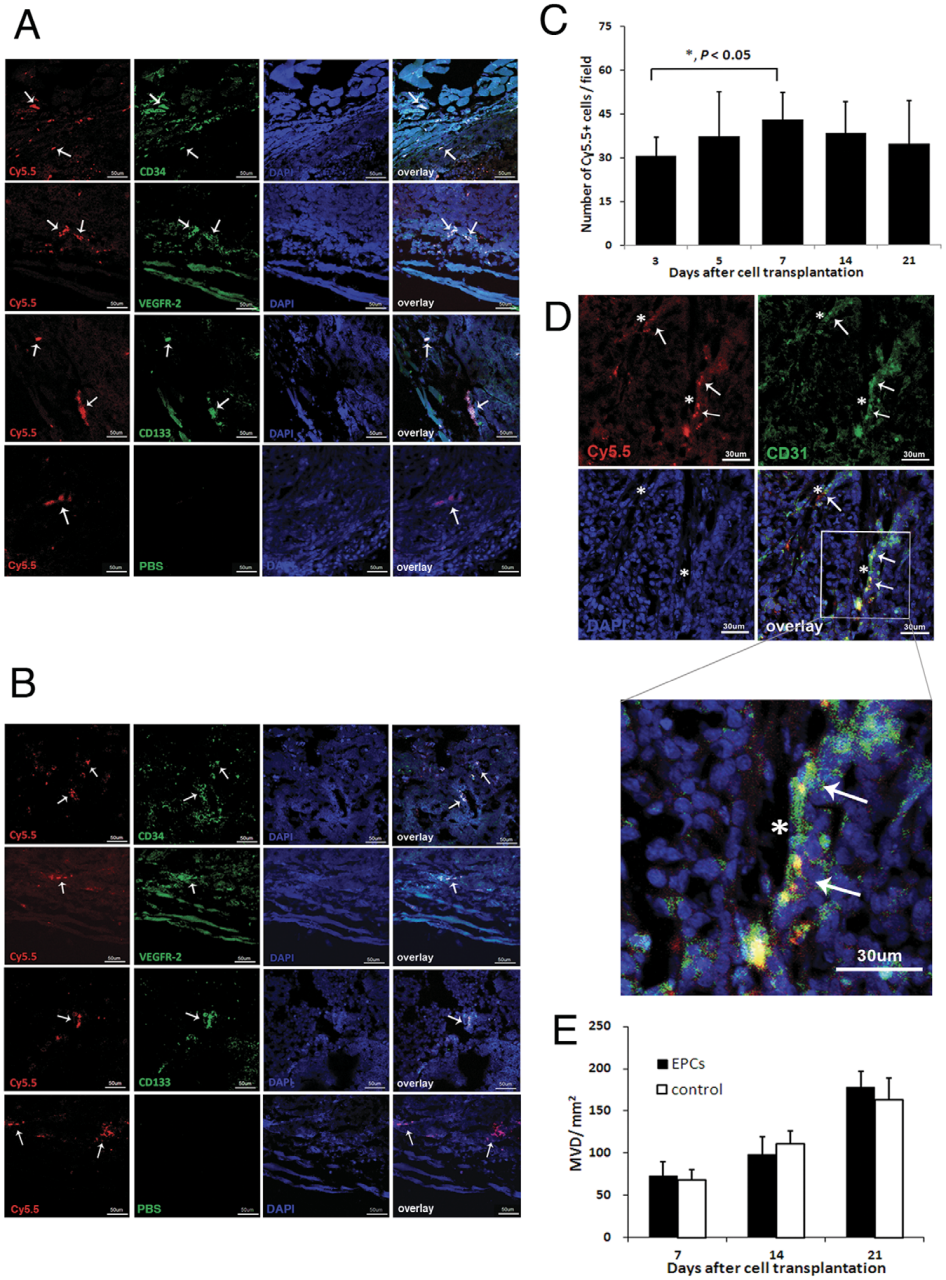
Histopathological examinations. Histopathological examinations were performed to validate imaging results. (A) Cy5.5 and anti-rat endothelial progenitor cell antibody positive cells (*arrow*) were observed in the connective tissue or at the tumor margin at day 3. Scale bar = 50 µm. In contrast, no Alex Fluo 488 signal was observed in the control group. (B) Sections collected at day 7 showed significant double positive cells (*arrow*) inside the tumor. Scale bar = 50 µm. (C) The numbers of Cy5.5 positive cells increased in tumors (30.75±6.24 cells/field of view at day 3 vs. 43.13±9.17 cells/field of view at day 7, *p*<0.05, *n* = 15), but remained the same afterwards. (D) CD31 and Cy5.5 double positive cells (*arrow*) were observed at the vessel walls inside the tumor at the day 14 (*asterisk* represent the lumen). Scale bar = 30 µm. (E) CD31 positive microvessels were counted in MVD quantification at 7, 14 and 21 days after cell transplantation. Compared to the control, no significant differences were found between the groups (*p*>0.05, *n* = 15).

Tumor sections obtained from group 1 were stained with anti-rat endothelial cell antibodies to localize the rat EPCs in tumor vasculature. Both Cy5.5 and CD34 positive cells were observed in tumors 3 days after EPCs transplantation, which indicate transcardiac transplanted EPCs incorporated to the vasculature. As expected, these transplanted EPCs located only in the connective tissues or at the margin of tumors at the early stage of tumor development and can be colocalized well with the CD133 and VEGFR-2 immunofluorescence (Fig. 5A). Sections collected at day 7 showed a significant amount of double positive cells (Fig. 5B), suggesting that a phenomenon of EPCs’ internal migration inside the tumor was observed. However, few transplanted EPCs were found inside the tumor at day 14. Even less Cy5.5 fluorescence was detected at day 21, possibly due to the dilution of the fluorescent probes over the period of the cell proliferation. To calculate the numbers of Cy5.5 positive cells, the fields of view were chosen at the rim of tumor sections with the concerns of heterogeneous distribution of transplanted EPCs at the early stages (Fig. 5C). The number of Cy5.5 positive cells increased in tumors (30.75±6.24 cells/FOV at day 3 vs. 43.13±9.17 cells/FOV at day 7, *p*<0.05, *n* = 15). It remained the same afterwards.

In order to investigate the origin of the new vessels, tumor sections obtained at day 7, 14 and 21 were stained for endothelial cell marker CD31. CD31 and Cy5.5 double positive cells were observed at the vessel walls inside the tumor at day 14 (Fig. 5D), which indicated the luminal incorporation of labeled cells. Morphologically, the vessel structure of each group was similar during the three week experiments. Some of the tumor vessels were tortuous and disorganized with loose EC connection. Neither severe abnormal vessel nor normalized one was observed after EPCs transplantation.

For microvessel density quantification, CD31 positive microvessels were counted at 7, 14 and 21 days after cell transplantation. Tumors in group 1 and 3 had similar MVD at day 7 (72.43±17.44 and 68.17±12.79/mm^2^, respectively). As tumors grew, the number of microvessels increased significantly in both groups (from 97.99±27.47 and 110.77±15.47/mm^2^ at day 14 to 177.99±19.11 and 163.31±25.66/mm^2^ at day 21). However, no statistically significant difference was found between the groups (*p*>0.05, *n* = 15, Fig. 5E).

### Gadolinium (Gd) Contents in Organs and Tumor Tissues

**Figure 6:**
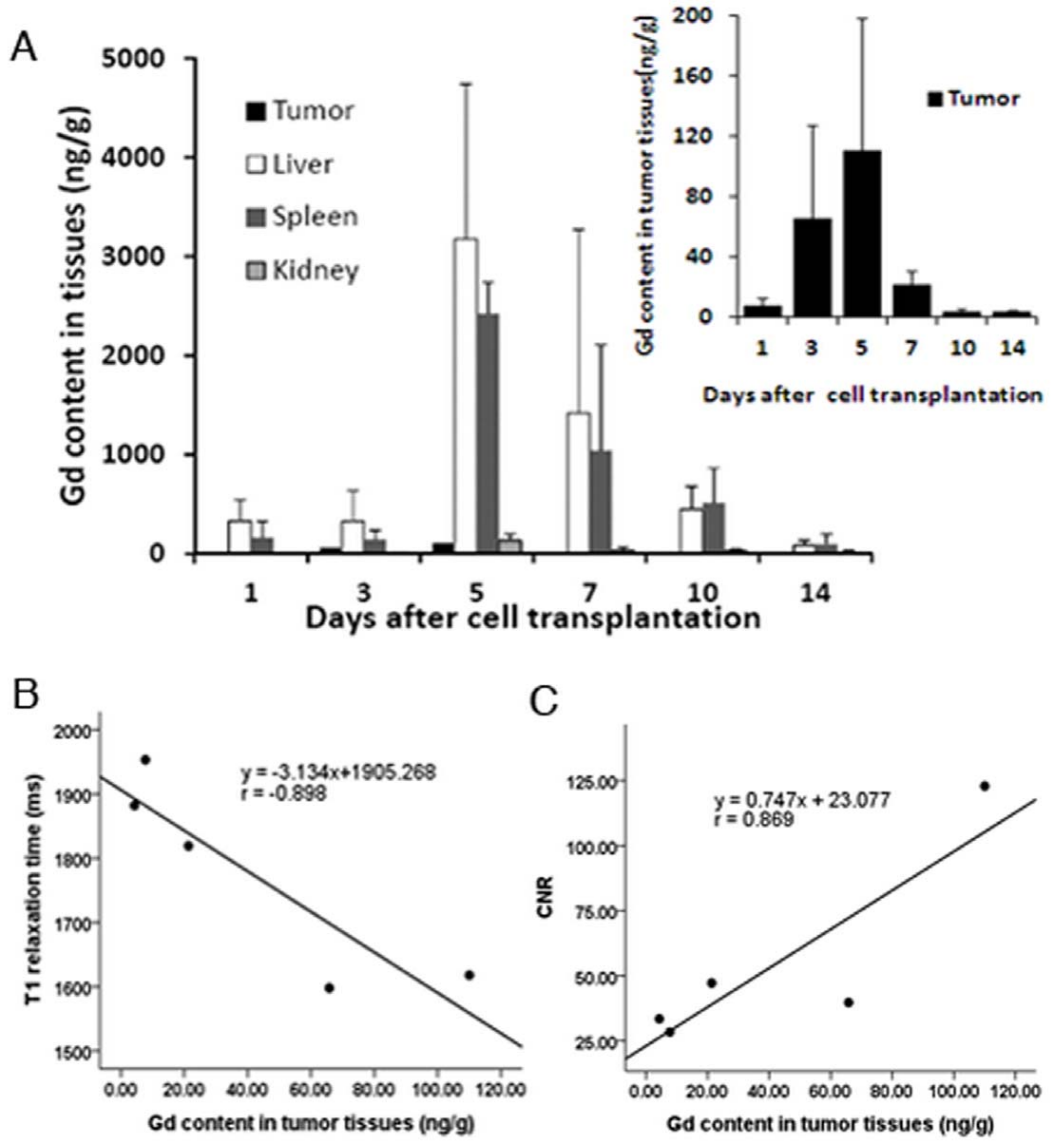
The amount of Gd in each organ. (A) The levels of Gd in the liver and spleen are much higher than that of tumor tissues; the concentration of Gd in the tumor reached peak at day 5. (B) A linear correlation (*r* = −0.898, *p*<0.05) was found between the averaged Gd content in the tumor and intratumoral T_1_ relaxation time. (C) The correlation between the averaged Gd content in the tumor and CNR of the tumor was significant (*r* = 0.869, *p*<0.05).

Quantitative analysis of tissue Gd content was performed using ICP-MS. Since Gd in normal tissues is not detectable, the amount of Gd in each organ provides the distribution of the Gd containing imaging probe quantitatively. Consistent with optical imaging, liver and spleen had much higher level of Gd among five organs tested, suggesting their phagocytosis to exogenous EPCs. Gd contents in tumors increased gradually at the different time points after transplantation of labeled EPCs. The peak concentration of 110.01±88.56 ng/g in the tumor was measured at day 5 (Fig. 6A). The amount of Gd in lungs as well as other tissues observed after day 14 was lower than 50 ng/g (data not shown). A linear correlation (*r* = −0.898, *p*<0.05, Fig. 6B) between the averaged Gd content in the tumor and intratumoral T_1_ relaxation time was found in group 1 at the different time points from day 1 to day 10. The correlation between averaged Gd content in the tumor and CNR in MRI was also significant (*r* = 0.869, *p*<0.05, Fig. 6C) at different time points from 1 to 10 days after transplantation of labeled EPCs. The linear correlation between Gd content of the tumor and T_1_ relaxation time or CNR supported the increase of the MR signal was the result of the homing of the Conjugate **1** labeled EPCs. As the tumors continued to grow, the Gd concentration was diluted leading to the intratumoral T_1_ relaxation time and CNR restored to the levels before the transplantation of labeled EPCs.

## Discussion

In this study, human breast cancer cell strain MDA-MB-231 was selected due to its high expression of VEGF, which involves in tumor neovascularization and facilitates homing of EPCs. Nolan and colleagues demonstrated that bone marrow derived EPCs incorporated into tumor neovasculature only at early stages of the tumor development as these cells were diluted with host-derived vessels afterward [4]. Moreover, the EPC homing can be interfered by active macrophages in organs like liver, spleen and lung. Therefore, it is suggested that EPCs should not be transplanted too early so that enough exogenous EPCs can be presented for imaging detection. In order to obtain high quality imaging and simulate the migrating activity of EPCs, the EPC transplantation in this study was chosen at 7 days after implantation of MDA-MB-231 cells. At this stage, the tumor was just formed with high demand for growing new vessels to support its growth.

Although intravenous injection has been widely used in conventional intravascular administration of experimental cells or agents, we selected intracardiac transplantation of EPCs by directly injecting cells into the left ventricle to reduce the phagocytosis by the liver and spleen in RES [29]. Even so, the actual amount of cells that home to the tumor in the current study was still low comparing to those captured in the liver and spleen. Results from optical imaging and quantitative analysis of Gd contents in different organs indicated that many labeled cells were phagocytosed by the liver. Therefore, the majority of transplanted cells were not able to reach the tumor site even if they were injected into the arterial system directly. This is an ineluctable difficulty that remains to be a major challenge in the research and development of EPC based imaging and therapies and needs to be addressed in the future. To overcome this problem, super selective injection of the transplanted cells into the tumor vessels, which is also convenient for clinical translation, might be an alternative.

It is noticed that the accumulation of Conjugate **1** labeled EPCs in the liver did not induce significant contrast enhancement on T_1_-weighted images. This is likely due to the homogenous distribution of labeled EPCs in the liver. In addition, the suboptimal detecting sensitivity of MRI may also lead to the indistinguishable signal enhancement. Perhaps the MR imaging efficiency of Conjugate 1 was not high enough even though its relaxivity was much higher than the commercial available contrast agent such as Gd^3+^-DOTA.

Both MR and optical imaging studies revealed the homing properties of transplanted EPCs. MRI was able to provide information on the spatial distribution of labeled EPCs in tumor tissues at high spatial resolution. Although T_2_-weighted MR contrast media, such as superparamagnetic iron oxide (SPIO) nanoparticles demonstrated high relaxivity and have been used for cell labeling and tracking [30]–[33], this approach is susceptible to the imaging artifacts from local field inhomogeneities. Paramagnetic Gd^3+^ chelates are capable of providing a positive contrast effect in T_1_-weighted imaging, which is less likely to be interfered by other non-tumor tissue and artifacts, such as necrosis, calcification, hemorrhage or metal deposition. In T_1_-weighted images obtained in this study, sparkles of hyperintense areas at the rim of the early stage tumors indicated the presence of homing cells. These signals quickly diminished when tumors grew to more than 1 cm in diameter (at about day 10), which may be explained as the result of the dilution of administered cells. The homing of labeled EPCs was directly confirmed by observation of the high level of Cy5.5 fluorescence from the probe in the tumors. It is noticed that optical signal enhancement was sensitive than that of MRI contrast enhancement. However, MRI is capable of multi-slice and 3D visualization of the tumor and distribution of the labeled cells. After all, the high correlation between the averaged fluorescent signal intensity in the tumor and intratumoral T_1_ relaxation time demonstrated the consistency of two imaging tracking modalities. Our study provides an example that the combination of two imaging methods and the use of a dual imaging probe allow for cross-validation of the experimental results and therefore, yield reliable findings.

Although Bagley [8] has demonstrated that co-injection of EPCs and MDA-MB-231 tumor cells resulted in an increased rate of the tumor growth, our results show that the transplantation of labeled EPCs did not alter the tumor size and VDT during the 3-week of experiments. The discrepancy between the results from this and previous studies may be due to the different observation time.

In the current study, rat origin EPCs were defined as CD34+/VEGFR2+/CD133+ cells during immunofluorescence analysis, while mature ECs were CD31+ cells. Although some of these markers are also used to define hematopoietic cell populations [34], there is no agreement with the specific marker of EPCs until recently. With capability of Cy5.5 fluorescence provided in our developed dual imaging probe, it is easy to differentiate rat origin EPCs/ECs from the host cells. Similar to the observations by MRI, immunofluorescence studies only found labeled EPCs at the margin of early stage tumors, likely due to hypervascularity associated with the rapid tumor growth. The number of Cy5.5 positive cells in tumor sections increased at the early stage of tumor growth, then were diluted because of their ingression and the increase of tumor volume. Although Cy5.5 positive cell counting may not be accurate to quantify the exact amount of transplanted EPCs in the tumor, it still can provide semi-quantitative information on the homing process of EPCs. Indeed, at the 14^th^ day of this study, some of the labeled ECs were found at the vessel walls, suggesting that the differentiation of EPCs into mature ECs may take place. Rat origin ECs were detected only in chimeric vessels with host origin ECs. This observation is similar to that reported by Nolan [4], which demonstrated luminal incorporation of rat origin ECs into existing host vessels. Similar to the tumor volume, MVD was not influenced by EPC transplantation within the time frame of this study (3 weeks). Taken together, the current research suggests that EPCs more likely play a role to promote angiogenesis rather than vasculogenesis [8].

As tumor blood vessels are structurally and functionally abnormal, tumor vascular normalization has been considered as a key step of antitumor therapy in recent studies [35]–[38] to block VEGF signaling in order to achieve vascular normalization. However, Bagley et al [8] confirmed that the blood vessels seem to be normalized while tumor cells were co-injected with EPCs and pericytes 29 days later. If this is true, EPCs may also become a possible marker for cancer diagnosis and treatment. In contrast to above reports, we did not find any structural change in tumor vessels after EPC transplantation. It is possible that some of the changes can be further detected by functional evaluation, such as vascular permeability analysis, since functional changes may appear earlier than those obtained from histopathological morphology examination [39]. The finding that EPC has no negative effect on tumor size or vasculature would potentially allow the development of genetically engineered EPCs to target tumor vasculature. Based on the successful transduction and use of AC133+ progenitor cells/EPCs to carry hNIS gene to the tumor site [40], [41], future researches can be designed to develop EPCs as gene carriers for other anti-tumor proteins. Therefore, genetically modified EPCs can be used both as targeting delivery vehicles and cellular probes tracked *in vivo* during cancer treatment.

### Conclusion

The current study used a novel paramagnetic/NIR fluorescence Conjugate **1** to label and track intracardiac transplanted EPCs in a xenograft tumor model. Imaging monitoring of the transplanted EPCs can be performed using MR and optical imaging for up to three weeks without altering tumor growth. Subsequent histopathological examination confirmed that EPCs were recruited to the tumor bed and then incorporated into new vessels. Tumor microvessel density was not affected by EPCs transplantation in the first three weeks of tumor development. These results demonstrated that EPCs play important roles in the development of endothelial cells during the tumor vascularization. The involvement and activities of EPCs can be investigated using molecular imaging approach non-invasively and longitudinally. Developed Conjugate **1** is a promising MRI and NIR dual imaging probe for *in vivo* tracking the EPCs in tumor models and for development of clinical applications of therapeutic transplantations of EPCs.

## Supporting Information

Table S1 **The experimental time point.**
(DOC)

Table S2 **The group designation.**
(DOCX)
