# Supplementary material for: Non-invasive Imaging of Endothelial Progenitor Cells in Tumor Neovascularization Using a Novel Dual-modality Paramagnetic/Near-Infrared Fluorescence Probe
Source: PLoS One. 2012 Nov 30;7(11):e50575. doi: 10.1371/journal.pone.0050575 (PMC3511537; doi:10.1371/journal.pone.0050575)
Supplement: Table S1 — The experimental time point. (DOC) [file pone.0050575.s001.doc]

**Table S1.** The experimental time point.

| Study | | Time Points | | | | | | | |
| --- | --- | --- | --- | --- | --- | --- | --- | --- | --- |
|  |  | Day 0 | Day 1 | Day 3 | Day 5 | Day 7 | Day 10 | Day 14 | Day 21 |
| Magnetic Resonance Imaging | T_1_-weighted image, T_2_-weighted image | n = 3 | n = 3 | n = 3 | n = 3 | n = 3 | n = 3 | n = 3 | n = 3 |
|  | T_1_-map | n = 3 | n = 3 | n = 3 | n = 3 | n = 3 | n = 3 |  |  |
| Tumor Volume | | n = 3 | n = 3 | n = 3 | n = 3 | n = 3 | n = 3 | n = 3 | n = 3 |
| Near Infrared Fluorescent Imaging | |  | n = 3 | n = 3 | n = 3 | n = 3 | n = 3 | n = 3 |  |
| Histopathology | Cy5.5+ Cell Counting |  |  | n = 15 | n = 15 | n = 15 |  | n = 15 | n = 15 |
|  | Microvessel Density |  |  |  |  | n = 15 |  | n = 15 | n = 15 |
| Gd Quantification | |  | n = 3 | n = 3 | n = 3 | n = 3 | n = 3 | n = 3 | n = 3 |
