# Supplementary material for: Non-invasive Imaging of Endothelial Progenitor Cells in Tumor Neovascularization Using a Novel Dual-modality Paramagnetic/Near-Infrared Fluorescence Probe
Source: PLoS One. 2012 Nov 30;7(11):e50575. doi: 10.1371/journal.pone.0050575 (PMC3511537; doi:10.1371/journal.pone.0050575)
Supplement: Table S2 — The group designation. (DOCX) [file pone.0050575.s002.docx]

**Table S2.** The group designation.

| Study | | Group | | |
| --- | --- | --- | --- | --- |
|  |  | Group 1 | Group 2 | Group 3 |
| Magnetic Resonance Imaging | T_1_-weighted image, T_2_-weighted image | ✓ | ✓ | ✓ |
|  | T_1_-map | ✓ | ✓ |  |
| Tumor Volume | | ✓ | ✓ | ✓ |
| Near Infrared Fluorescent Imaging | | ✓ | ✓ |  |
| Histopathology | Cy5.5+ Cell Counting | ✓ |  |  |
|  | Microvessel Density | ✓ |  | ✓ |
| Gd Quantification | | ✓ |  |  |
